# Duration of Anesthesia with Dexmedetomidine as Adjuvant to Ropivacaine in Supraclavicular Brachial Plexus Block: An Observational Study

**DOI:** 10.31726/jnma.8841

**Published:** 2024-12-31

**Authors:** Samyukta Acharya, Chitra Thapa, Nisha Shrestha, Pawan Kumar Hamal

**Affiliations:** 1 Department of Anesthesiology, Nepal Orthopedic Hospital, Jorpati, Kathmandu, Nepal; 2 Nepal Medical College, Jorpati, Kathmandu, Nepal; 3 National Academy of Medical Sciences, Mahaboudha, Kathmandu, Nepal

**Keywords:** *anesthesia*, *brachial plexus block*, *dexmedetomidine*, *ropivacaine*

## Abstract

**Introduction:**

Supraclavicular brachial plexus block is effective for upper limb surgeries, with ropivacaine offering prolonged action. Adding dexmedetomidine as an adjuvant may enhance block duration and quality. This study aimed to evaluate the mean duration of anesthesia with dexmedetomidine as an adjuvant to ropivacaine in supraclavicular brachial plexus blocks.

**Methods:**

An observational cross-section study was conducted among patients undergoing supraclavicular brachial plexus block for elective upper limb surgery in a tertiary care center. The block was performed using ultrasound-guided, single-injection technique with 0.25% ropivacaine and 0.5 ^g/kg dexmedetomidine. Sensory and motor block durations, along with analgesia, were assessed using standardized scales. A convenience sampling method was used. The point estimate was calculated at a 95% Confidence Interval. An ethical approval was taken from Institutional review Committee (Reference number: 026-077/078).

**Results:**

The mean duration of anesthesia was 592.42±137.73 minutes (548.62-636.21, 95% Confidence Interval). Sensory and motor block durations were 553.95±138.54 and 555.42±156.24 minutes, respectively, with median onset times of 15 (IQR: 5-22.5) and 24 (IQR: 17-30) minutes.

**Conclusions:**

The mean duration of anesthesia with dexmedetomidine as an adjuvant to ropivacaine in supraclavicular brachial plexus blocks was similar to other studies showing prolonged duration and accelerated sensory and motor block.

## INTRODUCTION

Regional anesthesia techniques, such as supraclavicular brachial plexus block, are commonly used for upper limb surgeries due to their effectiveness in providing surgical anesthesia and postoperative analgesia.^1,2^ Ropivacaine, a long-acting amide local anesthetic, is favored for its safety profile and prolonged action.^3^ Adding adjuvants like dexmedetomidine, a selective a_2_-adrenergic agonist, has shown a potential to enhance block duration and quality.^4,5^

Dexmedetomidine potentiates local anesthetics by stabilizing nerve membranes and promoting hyperpolarization, resulting in extended sensory and motor blockade.^6^ Exploring its clinical impact as an adjuvant to ropivacaine can help optimize dosing and improve outcomes, especially in procedures requiring prolonged pain relief.

The aim of this study was to determine the mean duration of anesthesia achieved by using dexmedetomidine as an adjuvant to ropivacaine in the supraclavicular brachial plexus block.

## METHODS

An observational cross-section study was conducted at Nepal Medical College Teaching Hospital (NMCTH), Jorpati, Kathmandu, Nepal which is a tertiary care centre. The data collection of the study was carried out for two months between October 2020 to December 2020. Institutional review Committee of NMCTH (Reference number: 026-077/078) provided approval before collecting the data. Written informed consent was obtained from all participants. Eligible participants were ASA (American Society of Anesthesiologists) physical status I and II patients scheduled for elective upper limb surgery under supraclavicular brachial plexus block. Patients with hypersensitivity to study drugs either ropivacaine or dexmedetomidine, pregnancy, lactation, hepatic, renal, or cardiopulmonary issues, long-term analgesic use, and local infection were excluded. Similarly, patients with inadequate sensory or motor block (>30 minutes after infiltration) or who required intraoperative analgesics, and patients with complications such as hypotension, bradycardia, Horner's syndrome, pneumothorax, or local anesthetic toxicity were excluded from the final analysis. A convenience sampling method was used. The sample size was calculated by using the following formula:


$$\begin{array}{ll} \text{n} & ={\text{Z}}^{\text{2}}\times \frac{{\left(\sigma \right)}^{\text{2}}}{{\text{e}}^{\text{2}}}\\ & =1.96^{2}\times \frac{310^{2}}{100^{2}}\\ & =37 \end{array}$$

where,

n = minimum required sample size
Z = 1.96 at 95% Confidence Interval (CI)
σ = standard deviation taken from published literature, 310^7^
e = margin of error

The minimum required sample size calculated was 37. However, 38 patients were enrolled during the study period.

Preanesthetic assessments were conducted one day before surgery. Anesthetic procedures and the Visual Analog Scale (VAS) were explained to the patients, and they were instructed to follow ASA fasting guidelines. In the operating room, baseline parameters including heart rate, blood pressure, and peripheral oxygen saturation were recorded. An 18-gauge intravenous cannula was inserted, and Ringer's lactate infusion was initiated. The supraclavicular brachial plexus block (BPB) was performed using ultrasound (USG)- guided, single-injection, nerve stimulator techniques by trained anesthesiologists with at least one year of experience in USG-guided BPB. All patients received 30 mL of a solution containing 0.25% ropivacaine and 0.5 μg/kg dexmedetomidine.

Sensory block was assessed using a 3-point scale.^8^

0: Normal sensation.

1: Loss of pinprick sensation.

2: Loss of touch sensation.

The duration of the sensory block was defined as the time from the onset of the complete sensory block (score 2) to the complete resolution of anesthesia (score 0).

Motor block was evaluated using the Modified Bromage Scale (MBS):^8^

0: Able to raise an extended arm to 90° for two seconds.

1: Able to flex the elbow and move fingers but unable to raise the arm.

2: Unable to flex the elbow but able to move fingers.

3: Unable to move the arm, elbow, or fingers.

The duration of the motor block was defined as the time from the onset of the complete motor block (MBS score 3) to recovery of full motor function (MBS score 0).

Block onset was assessed every 3 minutes until onset and then at 15, 30, 45, 60, 90, and 120 minutes. Subsequent assessments were conducted hourly until the block was completely resolved. Duration of analgesia was defined as the time from the onset of complete sensory block to the first request for rescue analgesia. Pain was assessed using the VAS (0 - 10). If the VAS score exceeded 3, 50 mg of intramuscular tramadol was administered, and the time was recorded.

Patients were monitored in the postoperative ward for 24 hours, with hourly assessments for pain, sensory recovery, and motor recovery. They were also asked to report subjective recovery of sensation and movement, which was verified by the investigator. The total amount of tramadol consumed in the first 24 hours was recorded.

Data were entered in Microsoft Excel 2019 and analyzed using SPSS Statistics for Windows, version 16.0 (SPSS Inc., Chicago, Ill., USA). A point estimate at 95% Confidence Interval was calculated.

## RESULTS

The average duration of anesthesia, from onset to complete resolution, was 592.42±137.73 minutes (548.62-636.21, 95% CI). The median duration of sensory anesthesia from onset to complete resolution was 553.95±138.54 minutes. The mean time from block administration to achieving complete sensory block was 15 (IQR: 5-22.5) minutes (Table 1).

**Table 1:** Duration of anesthesia and block achievement times (n=38).

Parameter	Mean±SD (minutes)
Duration of anesthesia	592.42±137.73
Sensory block duration	553.95±138.54
Motor block duration	555.42±156.24
**Median (IQR)**	
Onset of sensory block	15 (5-22.5)
Onset of motor block	24 (17-30)

The study included participants with a mean age of 36.37±14.04 years and a mean weight of 64.04±13.69 kg. The largest group of patients was in the 20-29 age range, comprising 11 patients (28.95%), followed by the 30-39 age range with 8 patients (21.05%) (Table 2). Regarding the sex distribution, 6 patients (15.79%) were male, and the remaining 32 patients (84.21%) were female.

**Table 2:** Age-wise distribution of patients (n=38).

Age Range (Years)	n (%)
10-19	6 (15.79)
20-29	11 (28.95)
30-39	8 (21.05)
40-49	6 (15.79)
50-59	5 (13.16)
60-69	2 (5.26)

## DISCUSSION

This study evaluated the duration of anesthesia when dexmedetomidine was used as an adjuvant to ropivacaine in a supraclavicular brachial plexus block. The results demonstrated a mean duration of anesthesia of 592.42±137.73 minutes, which is consistent with another study that reports a similar duration of around 590.2±40.5 minutes.^9^ Other studies conducted in India, reported a longer duration of anesthesia at 967.55±310.50 minutes^7^ and 805.7±205.^9^ minutes.^10^ However, another study showed shorter post-operative analgesia of 413.73±89.92 minutes as compared to our study.^11^ This could be attributed to differences in study protocols, doses of dexmedetomidine, and patient characteristics, highlighting the need for further research to standardize the combination of dexmedetomidine with ropivacaine for regional anesthesia.

The mean durations of sensory and motor anesthesia in our study were similar 553.95±138.54 and 555.42±156.24 minutes, respectively. These durations are notably longer than those reported in a similar study, which found motor block durations of 430.1±35.7 minutes and sensory block durations of 482.1±39.4 minutes.^9^ However, these durations were shorter compared to studies reporting sensory block durations of 789.45±187.72 minutes and 630.6±208.2 minutes, and motor block durations of 754.60±180.50 minutes and 545.9±224.0 minutes.^7,10^ However, the shorter motor block and sensory block duration of 312.0±49.91 and 379.40±55.09 minutes were also observed in another study.^11^ The discrepancy in block durations between studies could be influenced by factors such as the patient demographics, and regional variations in response to anesthesia.

The time to achieve a complete sensory block was shorter 15 (IQR: 5-22.5) compared to motor block onset 24 (IQR: 17-30), indicating a faster onset of sensory blockade in our study. These times are in line with studies that report longer onset times for motor blocks compared to sensory blocks, such as (18.75±6.37 vs 9.75±4.23 min)^7^ and (15.6±6.3 vs 9.5±5.8 minutes).^10^ Another study showed the onset times for motor slightly less than sensory blockages (7.5±2.3 vs 8.9±2.5 minutes) and is slower than in our study.^9^ These findings align with the hypothesis that dexmedetomidine prolongs the duration of anesthesia and enhances the quality of regional blocks.

The demographic characteristics showed a mean age of 36.37±14.04 years and a predominance of female participants (84.21%). The largest proportion of patients was in the 20-29 age range (28.95%), which may reflect the population distribution. These demographics might have contributed to the differences in the anesthetic effects of the drugs.

Dexmedetomidine, when added to ropivacaine, increases the duration of postoperative analgesia and sensory and motor block as well as reduces sufentanil use and the incidence of postoperative nausea and vomiting. Furthermore, it expedites the onset of sensory and motor block without increasing the risk of bradycardia or hypotension, highlighting its favorable safety profile.^12,13^ These findings are consistent with studies demonstrating similar benefits of dexmedetomidine as an adjuvant to ropivacaine in various regional anesthesia techniques, including tibial nerve blocks,^14^ epidural anesthesia for lower limb orthopedic procedures,^15^ pediatric lower abdominal surgeries,^16^ cesarean section^17^ and supraclavicular brachial plexus blocks for upper limb surgeries.^7,9^ The findings of this study suggest that dexmedetomidine, as an adjuvant to ropivacaine, prolongs the duration of anesthesia in supraclavicular brachial plexus blocks. The prolonged sensory and motor blockade duration could be beneficial in settings where extended pain relief is desired. However, the variability in onset and duration highlights the need for personalized dosing and careful monitoring. Compared with similar studies, the duration observed here is consistent with evidence supporting dexmedetomidine's role in enhancing local anesthetic effects.

Several limitations should be noted. Variability in individual response to anesthesia and potential confounding factors, such as differences in comorbidities or procedural techniques, were not fully controlled. The study's cross-sectional design precludes evaluation of long-term outcomes or adverse effects. Further research involving larger, more diverse populations is needed to confirm these findings and establish broader applicability. Additionally, multicenter studies could help address potential biases related to single-center procedural protocols and patient selection criteria.

## CONCLUSIONS

This study supports the use of dexmedetomidine as an adjuvant to ropivacaine in supraclavicular brachial plexus blocks, with prolonged anesthesia durations and faster sensory and motor block onset observed in our patient population similar to previously published articles. However, caution is warranted in extrapolating these findings to other settings without further validation.
